# The Doctor as a Missionary and Apostle of Hygiene

**Published:** 1910-05

**Authors:** 


### EDITORIAL DEPARTMENT.

### THE DOCTOR AS A MISSIONARY AND APOSTLE OF HYGIENE.

In every community the doctor stands out as an exceptional man. Generally, he is thought to be wise, moral and exemplary. I am sorry to say that there are exceptions. There is nothing more pitiful than to see a drunken doctor. At our State Association meetings I have more than once seen such sights. But the exception proves the rule. The doctor is looked up to, and can teach by precept and example. He should be a shining light. His is the opportunity to reach and teach the people in detail; hence he can be an important factor in the propaganda of reform,—the campaign of education now sweeping over the country. There is no use in trying to educate the masses by tracts and newspapers. The newspapers will not say a word against whiskey and patent medicines; it "injures business." The people must be spoken to, lectured to *en masse;* taught singly. Our only hope for reform in the matter of drinking and gonorrhea, the two most far-reaching and destructive evils of our civilization, lies in training the rising generation how to avoid their effects. In localities where it is held that "it will injure business" to shut up those hell-holes called "saloons" create in the minds of the boys a contempt and fear and disgust that will enable them to pass a saloon as they would avoid a smallpox patient and feel no kind of temptation to enter. "Who enters here leaves hope behind"; yea, more; he leaves health, happiness, heaven, behind. Boys should be made to appreciate the evils following even the "moderate" use of liquor, and the danger and disgrace of prostitution; and the family physician is the one to teach it. The thousands of family physicians can in this way lend efficient aid to those noble altruists who have taken the lead in organizing State and national societies for social hygiene, and it is the duty of every right-minded man to do so. All honor to Prince A. Morrow—the founder of it all. In this issue I have given considerable space to his teachings and I hope it will be heeded, and that every reader of the "Red Back" will feel it an imperative duty to cooperate with our colleagues at San Antonio who have just organized the Texas State Association of Hygiene. Let them, in season and out of season, in every household where their duties call them, preach to father, mother, brother, sister, uncle, aunt and grandfather the gospel of pure lives—sober, clean, useful lives,—and in two more generations the world will see fewer lunatics, fewer criminals, fewer mutilated women cut for septic salpingitis, fewer blind, deaf and dumb, fewer unhappy households, prostitutes, divorces; hear less wail of miserable wives and cry of hungry children. The doctor should teach his patrons that alcohol is a deadly narcotic, and that Peruna is a disguised alcoholic. That Mrs. Winslow's Soothing Syrup contains morphine. That "Duffey's Pure Malt Whiskey" is a delusion, a fraud, and that the medical profession *do not* endorse it; that it *does not* produce refreshing sleep, as advertised, but locks one in the deadly embrace of a poison-stupor. That the advertised female regulators produce abortion. That gonorrhea is a filth disease to be shunned like any other pestilence. In fact, let all of my readers take Dr. Morrow's article in this issue for his Bible and preach it in every household. Only by some such means can the rising generation be reached and influenced.

In addition to this the doctor can incidentally warn his patrons, especially in the country, of the danger of starting a fire with coal oil; of carbolic acid and concentrated lye; of fireworks and toy pistols, and acquaint them with the true nature and deadly mission of the house fly. In brief, the doctor can teach his patrons how to not be sick. I commend to my readers the work done and being done by the societies above mentioned, and a careful reading of the articles in this issue. Let the doctors also tell the people that "night air" is not dangerous, that shut rooms are, and that we can not have too much fresh air nor too much fresh water inside and out; that a cold bath is a powerful tonic, and instruct them in the importance of bodily cleanliness as a preventive of many diseases. And may God prosper the doctor in such work.

